# The association between depressive symptoms and limitations in disability domains among US adults

**DOI:** 10.1016/j.xjmad.2024.100103

**Published:** 2024-12-30

**Authors:** Shakila Meshkat, Qiaowei Lin, Vanessa K. Tassone, Reinhard Janssen-Aguilar, Wendy Lou, Venkat Bhat

**Affiliations:** a Interventional Psychiatry Program, St. Michael’s Hospital, Toronto, Ontario, Canada; b Department of Biostatistics, Dalla Lana School of Public Health, University of Toronto, Toronto, Ontario, Canada; c Neuroscience Research Program, St. Michael’s Hospital, Toronto, Ontario, Canada; d Department of Psychiatry, University of Toronto, Toronto, Ontario, Canada; e Institute of Medical Science, Temerty Faculty of Medicine, University of Toronto, Toronto, Ontario, Canada

**Keywords:** Depression, Disability, NHANES, Cross-sectional study

## Abstract

This study aims to evaluate the association of depressive symptoms and their severity with overall disability and disability domains, and to assess temporal changes in these associations. Data from the National Health and Nutrition Examination Survey (NHANES) 2013–2018 were analyzed. A total of 15,565 participants were included, with 1383 (8.19 %) reporting depressive symptoms. Depressive symptoms were strongly associated with overall disability (aOR = 7.82; 95 % CI = 6.27, 9.75; p < 0.001) and with six specific domains (aOR = 2.54 for hearing, 2.84 for seeing, 10.53 for concentrating, 4.42 for walking, 6.01 for dressing or bathing, and 7.36 for doing errands alone; all p < 0.001). Each unit increase in PHQ-9 score was linked to a 21 % increase in the odds of overall disability (aOR = 1.21; 95 % CI = 1.19, 1.23; p < 0.001) and to a 9 %-25 % increase in the odds of domain-specific difficulties. Both cognitive-affective (aOR = 1.35; 95 % CI = 1.30, 1.40) and somatic scores (aOR = 1.36; 95 % CI = 1.33, 1.39) were associated with overall disability and domain-specific difficulties, with each unit increase linked to 13 %-45 % higher odds. Temporal analysis indicated that depressive symptoms are linked to a higher prevalence of disability, with this association persisting and slightly increasing over time. Our findings indicated the association between depressive symptoms and disability. Future studies should further replicate our results and evaluate the mechanisms underlying this phenomenon.

## 1 Introduction

Depression is a chronic mood disorder that affects 280 million individuals globally [1]. It is characterized by persistent sadness, anhedonia, low self-esteem, and a range of physical and cognitive symptoms that can impair daily functioning [2]. Depressive symptoms can vary in intensity, ranging from mild to severe [3]. Depression is increasingly recognized as a multifaceted disorder, comprising two primary symptom clusters: somatic and cognitive-affective [4]. Rather than a single, homogeneous condition, this bifurcation underscores the heterogeneous nature of depression [5]. Concurrently, the cognitive-affective cluster includes psychological symptoms such as anhedonia, pervasive low mood, feelings of guilt, impaired concentration, and suicidal ideation [5]. Somatic symptoms impact different aspects such as sleep, energy, appetite, and psychomotor slowing or restlessness [5]. Notably, cognitive-affective symptoms are strongly linked to perceived unmet psychological care needs, distinguishing them from somatic manifestations [5]. This delineation is supported by research suggesting that separate analysis of these symptom clusters provides a more refined understanding of depression, particularly in relation to health outcomes, than assessments based solely on global depressive symptom severity [6].

Disability is defined as a loss or restriction of functional ability or activity as a result of impairment of the body or mind [7]. Disability and depression are critical public health concerns that often coexist, creating a substantial burden for individuals and society [8]. Depression, a leading cause of disability worldwide, is associated with significant impairments across multiple life domains [9]. The relationship between depression and disability is complex, with depressive symptoms potentially exacerbating difficulties in daily functioning, while disability itself can increase the risk of depression through factors such as social isolation, reduced physical activity, and loss of independence [9]. Research indicates that there is a bidirectional relationship between depression and disability, with either initiating the worsening of present symptoms [10]. Currently, it has been found that individuals with disabilities experience various factors that contribute to depression, including mobility limitations, accessibility challenges, social barriers, isolation, employment difficulties, and health issues [11]. Moreover, current studies indicate a correlation between depression and disability suggesting that those who suffer from disabilities are more likely to develop depression [12].

The extent to which depressive symptoms influence specific domains of disability remains underexplored. Understanding these associations is essential for developing targeted interventions that address both mental health and functional limitations. Moreover, the magnitude of the depressive symptom-disability association and temporal changes remain undetermined. Therefore, in this study, we aim to evaluate the association of depressive symptoms, symptom severity, and symptom clusters with overall disability and specific domains. We will also assess temporal changes in these associations.

## 2 Methods

### 2.1 Study population

The study analyzed data from the National Health and Nutrition Examination Survey (NHANES) spanning 2013–2018. NHANES is a program of studies designed to assess the health and nutritional status of adults and children in the United States, by creating cross-sectional surveys that combine both physical examinations and interviews. The interview questionnaire includes demographic, socioeconomic, dietary, and health-related questions. In contrast, the physical examination consists of medical, dental, and physiological measurements along with laboratory tests administered by trained healthcare professionals. The participants are chosen through a statistical process. NHANES selects individuals from the United States based on region, neighborhood, and household. Each year, one or two neighborhoods from each region are selected. Within each designated neighborhood, a substantial number of households are selected. From this large sample of households, a smaller sample is selected. Residents are asked to complete a survey regarding the members of the household, and a statistical computer software determines their eligibility to participate in NHANES.

In this study, we limited the analysis to participants aged 18 and older who completed both the Mental Health - Depression Screener (DPQ) and the Disability Questionnaire (DLQ). Additional information on NHANES methodology can be found on the CDC website (https://wwwn.cdc.gov/nchs/nhanes/analyticguidelines.aspx#)

### 2.2 Exposure variable

The Patient Health Questionnaire-9 (PHQ-9) was used to assess depressive symptoms. The questionnaire includes nine items that assess the prevalence of depressive symptoms in the last two weeks. A follow-up question assesses the overall impairment of the depressive symptoms. Scores range from 0 to 3, or "not at all," "several days," "more than half the days" and "nearly every day". The total scores were calculated and could range from 0 to 27. A score of 10 or higher has been suggested to indicate the presence of depressive symptoms. The questions were administered using the Computer-Assisted Personal Interviewing (CAPI) system. The PHQ-9 is widely recognized for its reliability and validity. Its scores can be divided into cognitive-affective (items 1, 2, 6, 7, and 9) and somatic (items 3, 4, 5, and 8) symptom clusters, both of which were treated as continuous variables in the present analysis.

### 2.3 Outcome variable

Disability was evaluated using the DLQ. We identified participants with disabilities in six specific domains, including hearing, seeing, concentrating, walking, dressing or bathing, and doing errands alone, based on a 'Yes' response to the questions DLQ010, DLQ020, DLQ040, DLQ050, DLQ060, and DLQ080, respectively. Participants who answered 'No' to these questions were considered as not having difficulties in the corresponding domains. For overall disability, participants were classified as having a disability if they answered 'Yes' to any of the questions mentioned above. Those who reported no difficulties across all domains were considered as having no overall disability.

### 2.4 Covariates

To control for potential confounding variables in the relationship between depressive symptoms and disability, the following covariates were incorporated into the analysis: age, sex, race, family income to poverty ratio (PIR), education level, body mass index (BMI), marital status, and medical histories of diabetes, hypertension (HTN), stroke, coronary heart disease (CHD), and cancer. Age was treated as a continuous variable, while sex was categorized as either male or female. Race was divided into five groups: Mexican American, other Hispanic, non-Hispanic White, non-Hispanic Black, and other/multiracial. PIR was classified into low income (≤1.3) and middle-to-high income (>1.3). Education was grouped into four categories: less than high school, high school or equivalent, some college or Associate of Arts degree, and college graduate or higher. BMI was categorized into underweight (<18.5 kg/m²), normal weight (18.5 to ≤24.9 kg/m²), overweight (25 to ≤29.9 kg/m²), and obese (≥30 kg/m²). Marital status was classified as never married, married, widowed, divorced, separated, or living with a partner.

Participants were considered to have diabetes if they answered "yes" to the question DIQ010, which asked, "Other than during pregnancy, has a medical professional ever told you that you have diabetes or sugar diabetes?" Those who answered "no" or "borderline" were classified as non-diabetic. A history of HTN was identified by affirmative answers to both BPQ020 ("Have you ever been diagnosed with hypertension or high blood pressure by a healthcare provider?") and BPQ030 ("Were you informed on two or more separate occasions that you had hypertension or high blood pressure?"). Stroke history was determined by a "yes" response to MCQ160F ("Has a doctor or health professional ever told you that you had a stroke?"). CHD was classified based on a "yes" answer to MCQ160C ("Has a doctor or health professional ever told you that you had coronary heart disease?"). Participants who answered "yes" to MCQ220 ("Has a doctor or health professional ever told you that you had cancer or any kind of malignancy?") were categorized as having a history of cancer. Adjusted models included all covariates, while unadjusted models only included the exposure variables of interest.

The choice of covariates was guided by their potential to influence the relationship between depressive symptoms and disability. We selected covariates based on their established associations with both depression and disability and their consistent availability in the NHANES datasets analyzed. These conditions are commonly assessed in NHANES and provide reliable data across survey years. While other conditions are also linked to depressive symptoms, they were excluded due to inconsistent data collection in NHANES, which would result in substantial missing data. Including variables with high levels of missing data could reduce statistical power or introduce bias, and therefore, we prioritized covariates with robust and complete datasets.

### 2.5 Statistical analysis

Analyses were performed in R Studio (v 4.4.0) using the 'survey' package to account for survey weights. The ID variable SDMVPSU and strata variable SDMVSTRA were applied, and MEC survey weights were adjusted by dividing by three (WTMEC2YR/3) to account for the combination of three survey cycles. Survey weights are critical for ensuring that the study findings are representative of the broader U.S. population. They adjust for the complex sampling design of NHANES, including oversampling of certain subpopulations and non-response bias. This ensures that the estimates are not biased and reflect the population-level trends accurately. In the table summarizing demographic characteristics, unweighted frequencies were generated using the CreateTableOne() function, while weighted percentages were calculated with the svyCreateTableOne() function. Categorical variables were presented as raw frequencies and weighted percentages, whereas continuous variables were reported as weighted means and standard deviations (SD). Differences in categorical demographic characteristics between groups with and without depressive symptoms were evaluated using a chi-square test of independence (p-value ≤ 0.05), while a *t*-test was used for continuous variables.

Multivariable logistic regression was used to assess the relationships of depressive symptoms (yes/no), depressive symptom severity (total PHQ-9 score), and somatic and cognitive-affective cluster scores with overall disability and specific domains of disability. Variance inflation factors (VIF) were used to assess multicollinearity, with all values being less than 4. To ensure the robustness of the results, a sensitivity analysis was performed. PHQ-8 scores were created by excluding the item related to concentration difficulties, specifically: 'Over the last 2 weeks, how often have you been bothered by trouble concentrating on things, such as reading the newspaper or watching TV?' This analysis aimed to assess whether the association between depressive symptom severity and impairments in concentration remained strong after removing this specific item. To investigate temporal trends in the prevalence of overall disability, we stratified the sample by the presence or absence of depressive symptoms. Using the survey design weights in NHANES, we calculated mean prevalence estimates and associated standard errors for each survey cycle (via the svyby function). We then plotted these estimates as a line chart to display the observed prevalence at each cycle and added smoothed trend lines (geom_smooth) to help visualize patterns over time. Because each survey cycle is an independent cross-section, the smoothed lines should be interpreted only as a descriptive illustration of changes in prevalence between cycles, rather than as true longitudinal trajectories. We opted not to perform imputation for the missing survey data to avoid introducing potential bias or inaccuracies. Thus, missing data were handled using listwise deletion, excluding cases with any missing values for variables in the model from the analysis.

## 3 Results

### 3.1 Study sample

**Fig. 1:**
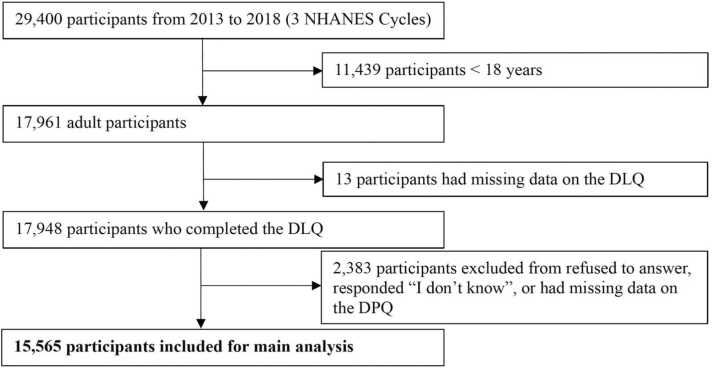
Participant inclusion flowchart. Abbreviations: NHANES = National Health and Nutrition Examination Survey; DLQ = Disability Questionnaire; DPQ = Mental Health - Depression Screener Questionnaire.

Data were gathered from 29,400 individuals over three cycles between 2013 and 2018. The inclusion criteria for participants are detailed in Fig. 1. The final sample consisted of 15,565 individuals, with 1383 (8.19 %) experiencing depressive symptoms. Of the total participants, 4230 (23.78 %) reported overall disability limitations, of which 1248 (7.18 %) had hearing difficulties, 966 (4.94 %) had seeing difficulties, 1598 (9.45 %) had trouble concentrating, 2190 (11.16 %) had walking difficulties, 780 (3.87 %) experienced challenges with dressing or bathing, and 1174 (6.29 %) reported difficulty doing errands alone. Among those with overall disabilities, 951 had depressive symptoms. The mean age of the participants was 47.09 years (SD = 17.65), and 7998 (51.37 %) were female. Table 1 outlines the demographic characteristics of the sample, stratified by the presence of depressive symptoms, along with any significant differences in the covariates.

**Table 1:** Demographic characteristics, stratified by presence of depressive symptoms (n = 15,565).

Characteristic	Total	Depressive symptoms - No	Depressive symptoms - Yes	*p*-value
Sample Size	15,565	14,182	1383	-
Total PHQ−9 scores (mean (SD))	3.13 (4.12)	2.16 (2.41)	13.98 (3.79)	< 0.001
Cognitive-Affective scores (mean (SD))	1.22 (2.23)	0.72 (1.25)	6.80 (3.00)	< 0.001
Somatic scores (mean (SD))	1.91 (2.31)	1.44 (1.64)	7.17 (2.18)	< 0.001
Disability (%)				< 0.001
No Yes	11,335 (76.22) 4230 (23.78)	10,903 (80.06) 3279 (19.94)	432 (33.17) 951 (66.83)	
Hearing (%)				< 0.001
No Yes	14,315 (92.82) 1248 (7.18)	13,159 (93.49) 1022 (6.51)	1156 (85.31) 226 (14.69)	
Seeing (%)				< 0.001
No Yes	14,597 (95.06) 966 (4.94)	13,453 (95.95) 728 (4.05)	1144 (85.11) 238 (14.89)	
Concentrating (%)				< 0.001
No Yes	13,965 (90.55) 1598 (9.45)	13,201 (93.75) 979 (6.25)	764 (54.65) 619 (45.35)	
Walking (%)				< 0.001
No Yes	13,371 (88.84) 2190 (11.16)	12,554 (90.95) 1624 (9.05)	817 (65.22) 566 (34.78)	
Dressing or bathing (%)				< 0.001
No Yes	14,784 (96.13) 780 (3.87)	13,665 (97.27) 516 (2.73)	1119 (83.38) 264 (16.62)	
Doing errands alone (%)				< 0.001
No Yes	14,383 (93.71) 1174 (6.29)	13,429 (95.80) 748 (4.20)	954 (70.28) 426 (29.72)	
Age (mean (SD))	47.09 (17.65)	47.10 (17.68)	47.01 (17.33)	0.911
Sex (%)				< 0.001
Male Female	7567 (48.63) 7998 (51.37)	7052 (49.76) 7130 (50.24)	515 (36.01) 868 (63.99)	
Race (%)				0.271
Mexican American Non-Hispanic Black Non-Hispanic White Other Hispanic Other Race - Including Multi-Racial	2379 (9.13) 3358 (11.12) 5814 (64.74) 1597 (6.15) 2417 (8.86)	2172 (9.21) 3069 (11.05) 5252 (64.88) 1422 (6.05) 2267 (8.81)	207 (8.18) 289 (11.94) 562 (63.18) 175 (7.30) 150 (9.40)	
PIR (%)				< 0.001
Low Income Mid-to-High Income	4530 (22.00) 9509 (78.00)	3914 (20.48) 8892 (79.52)	616 (39.17) 617 (60.83)	
Education (%)				< 0.001
Less than high school High school graduate/ GED Some college/ AA degree College graduate or above	3065 (12.98) 3377 (23.43) 4629 (32.22) 3679 (31.37)	2647 (12.22) 3056 (23.12) 4205 (31.91) 3522 (32.75)	418 (21.51) 321 (26.85) 424 (35.74) 157 (15.90)	
BMI (%)				< 0.001
Healthy weight Underweight Overweight Obese	4158 (26.93) 255 (1.54) 4879 (31.63) 6109 (39.89)	3869 (27.22) 224 (1.47) 4545 (32.34) 5402 (38.97)	289 (23.73) 31 (2.34) 334 (23.68) 707 (50.25)	
Marital Status (%)				< 0.001
Never married Married Widowed Divorced Separated Living with partner	2722 (18.31) 7500 (54.64) 1080 (5.69) 1665 (10.23) 503 (2.46) 1280 (8.67)	2446 (18.02) 7033 (56.28) 942 (5.40) 1424 (9.58) 421 (2.25) 1163 (8.48)	276 (21.65) 467 (36.28) 138 (8.89) 241 (17.56) 82 (4.84) 117 (10.79)	
Diabetes (%)				< 0.001
No Yes	13,403 (89.34) 2152 (10.66)	12,313 (89.81) 1862 (10.19)	1090 (83.97) 290 (16.03)	
HTN (%)				< 0.001
No Yes	11,065 (73.85) 4453 (26.15)	10,272 (75.01) 3867 (24.99)	793 (60.75) 586 (39.25)	
Stroke (%)				< 0.001
No Yes	14,176 (97.11) 563 (2.89)	12,975 (97.47) 448 (2.53)	1201 (93.12) 115 (6.88)	
CHD (%)				< 0.001
No Yes	14,077 (96.22) 631 (3.78)	12,874 (96.56) 519 (3.44)	1203 (92.37) 112 (7.63)	
Cancer (%)				0.896
No Yes	13,272 (88.66) 1482 (11.34)	12,108 (88.68) 1323 (11.32)	1164 (88.48) 159 (11.52)	

Abbreviation: PHQ-9 = nine-item Patient Health Questionnaire; PIR = Ratio of Family Income to Poverty; BMI = Body Mass Index; HTN = Hypertension; CHD = Coronary Heart Disease; SD = Standard Deviation.

Categorical characteristics reported as unweighted frequency and weighted percent.

Continuous characteristics reported as weighted mean and standard deviation.

*P*-values < 0.05 denote significant differences between depressive symptoms (yes/no).

### 3.2 Depressive symptoms and overall disability

The findings demonstrated a positive association between depressive symptoms and overall disability limitations (Table 2). Individuals exhibiting depressive symptoms had 7.82 times the odds of reporting overall disability (aOR = 7.82; 95 % CI = 6.27, 9.75; p < 0.001). In addition, each one-point increase in the PHQ-9 score corresponded to a 21 % higher likelihood of experiencing overall disability (aOR = 1.21; 95 % CI = 1.19, 1.23; p < 0.001).

**Table 2:** Main effects models for the associations of presence of depressive symptoms and total PHQ-9 scores with overall disability.

**Depressive symptoms - yes**				
	OR (95 % CI)	*p*-value	aOR (95 % CI)	*p*-value
Overall Disability	8.09 (6.74,9.71)	**< 0.001**	7.82 (6.27,9.75)	**< 0.001**
**PHQ−9 Scores**				
	OR (95 % CI)	*p*-value	aOR (95 % CI)	*p*-value
Overall Disability	1.22 (1.20,1.23)	**< 0.001**	1.21 (1.19,1.23)	**< 0.001**

Note: OR = unadjusted odds ratio; aOR = adjusted odds ratio; CI = confidence interval; the reference level for depressive symptoms is “no”; p-values < 0.05 denote statistical significance.

### 3.3 Depressive symptoms and each domain of disability

The results indicated a positive association between depressive symptoms and difficulties in various domains of disability (Table 3). Individuals with depressive symptoms had higher odds of experiencing various difficulties compared to those without depressive symptoms: 2.54 times higher for hearing (aOR = 2.54; 95 % CI = 1.88, 3.43; p < 0.001), 2.84 times higher for seeing (aOR = 2.84; 95 % CI = 2.20, 3.67; p < 0.001), 10.53 times higher for concentrating (aOR = 10.53; 95 % CI = 8.49, 13.05; p < 0.001), 4.42 times higher for walking (aOR = 4.42; 95 % CI = 3.52, 5.54; p < 0.001), 6.01 times higher for dressing or bathing (aOR = 6.01; 95 % CI = 4.30, 8.40; p < 0.001), and 7.36 times higher for doing errands alone (aOR = 7.36; 95 % CI = 5.82, 9.32; p < 0.001).

**Table 3:** Main effects models for the associations of presence of depressive symptoms and total PHQ-9 scores with each domain of disability.

**Depressive symptoms - yes**				
**Disability Domain**	OR (95 % CI)	*p*-value	aOR (95 % CI)	*p*-value
Hearing	2.47 (1.90,3.22)	**< 0.001**	2.54 (1.88,3.43)	**< 0.001**
Seeing	4.15 (3.35,5.13)	**< 0.001**	2.84 (2.20,3.67)	**< 0.001**
Concentrating	12.45 (10.45,14.84)	**< 0.001**	10.53 (8.49,13.05)	**< 0.001**
Walking	5.36 (4.59,6.27)	**< 0.001**	4.42 (3.52,5.54)	**< 0.001**
Dressing or bathing	7.09 (5.51,9.14)	**< 0.001**	6.01 (4.30,8.40)	**< 0.001**
Doing errands alone	9.64 (8.09,11.49)	**< 0.001**	7.36 (5.82,9.32)	**< 0.001**
**PHQ−9 Scores**				
**Disability Domain**	OR (95 % CI)	*p*-value	aOR (95 % CI)	*p*-value
Hearing	1.08 (1.07,1.10)	**< 0.001**	1.09 (1.07,1.11)	**< 0.001**
Seeing	1.13 (1.12,1.15)	**< 0.001**	1.11 (1.08,1.13)	**< 0.001**
Concentrating	1.26 (1.24,1.28)	**< 0.001**	1.25 (1.22,1.28)	**< 0.001**
Walking	1.17 (1.15,1.18)	**< 0.001**	1.15 (1.13,1.17)	**< 0.001**
Dressing or bathing	1.18 (1.16,1.20)	**< 0.001**	1.17 (1.14,1.20)	**< 0.001**
Doing errands alone	1.22 (1.20,1.24)	**< 0.001**	1.20 (1.17,1.22)	**< 0.001**

Note: OR = unadjusted odds ratio; aOR = adjusted odds ratio; CI = confidence interval; the reference level for depressive symptoms is “no”; p-values < 0.05 denote statistical significance.

Furthermore, a unit increase in PHQ-9 score was associated with a 9 % increase in the odds of having difficulties in hearing (aOR = 1.09; 95 % CI = 1.07, 1.11; p < 0.001), an 11 % increase in the odds of having difficulties in seeing (aOR = 1.11; 95 % CI = 1.08, 1.13; p < 0.001), a 25 % increase in the odds of having difficulties in concentrating (aOR = 1.25; 95 % CI = 1.22, 1.28; p < 0.001), a 15 % increase in the odds of having difficulties in walking (aOR = 1.15; 95 % CI = 1.13, 1.17; p < 0.001), a 17 % increase in the odds of having difficulties in dressing or bathing (aOR = 1.17; 95 % CI = 1.14, 1.20; p < 0.001), and a 20 % increase in the odds of having difficulties in doing errands alone (aOR = 1.20; 95 % CI = 1.17, 1.22; p < 0.001).

Table S1 presents the results of the sensitivity analysis conducted using PHQ-8 scores. The findings from the sensitivity analysis did not alter the conclusions.

### 3.4 Cognitive-affective and somatic scores and overall disability

A positive association was observed between cognitive-affective and somatic scores and overall disability (Table 4). Each one-point increase in cognitive-affective scores was associated with a 35 % higher likelihood of overall disability (aOR = 1.35; 95 % CI = 1.30, 1.40; p < 0.001), while a one-point increase in somatic scores corresponded to a 36 % higher likelihood (aOR = 1.36; 95 % CI = 1.33, 1.39; p < 0.001).

**Table 4:** Main effects models for the associations of cognitive-affective and somatic scores with overall disability.

**Cognitive-Affective Scores**				
	OR (95 % CI)	*p*-value	aOR (95 % CI)	*p*-value
Overall Disability	1.37 (1.33,1.41)	**< 0.001**	1.35 (1.30,1.40)	**< 0.001**
**Somatic Scores**				
	OR (95 % CI)	*p*-value	aOR (95 % CI)	*p*-value
Overall Disability	1.37 (1.35,1.40)	**< 0.001**	1.36 (1.33,1.39)	**< 0.001**

Note: OR = unadjusted odds ratio; aOR = adjusted odds ratio; CI = confidence interval; p-values < 0.05 denote statistical significance.

### 3.5 Cognitive-affective and somatic scores and each domain of disability

Cognitive-affective and somatic scores were positively associated with difficulties across all disability domains (Table 5). For cognitive-affective scores, the strongest association was with difficulties in concentrating (aOR = 1.45; 95 % CI = 1.39, 1.51). Similarly, for somatic scores, the strongest association was also with difficulties in concentrating (aOR = 1.42; 95 % CI = 1.37, 1.47). Detailed odds ratios, confidence intervals, and p-values for each domain are presented in Table 5.

**Table 5:** Main effects models for the associations of cognitive-affective and somatic scores with each domain of disability.

**Cognitive-Affective Scores**				
**Disability Domain**	OR (95 % CI)	*p*-value	aOR (95 % CI)	*p*-value
Hearing	1.13 (1.10,1.16)	**< 0.001**	1.13 (1.09,1.16)	**< 0.001**
Seeing	1.22 (1.19,1.25)	**< 0.001**	1.17 (1.13,1.21)	**< 0.001**
Concentrating	1.49 (1.44,1.54)	**< 0.001**	1.45 (1.39,1.51)	**< 0.001**
Walking	1.25 (1.23,1.28)	**< 0.001**	1.21 (1.18,1.25)	**< 0.001**
Dressing or bathing	1.28 (1.24,1.32)	**< 0.001**	1.25 (1.21,1.30)	**< 0.001**
Doing errands alone	1.36 (1.32,1.40)	**< 0.001**	1.31 (1.27,1.36)	**< 0.001**
**Somatic Scores**				
**Disability Domain**	OR (95 % CI)	*p*-value	aOR (95 % CI)	*p*-value
Hearing	1.16 (1.12,1.19)	**< 0.001**	1.17 (1.13,1.21)	**< 0.001**
Seeing	1.24 (1.21,1.28)	**< 0.001**	1.19 (1.15,1.24)	**< 0.001**
Concentrating	1.46 (1.42,1.49)	**< 0.001**	1.42 (1.37,1.47)	**< 0.001**
Walking	1.34 (1.32,1.37)	**< 0.001**	1.31 (1.27,1.35)	**< 0.001**
Dressing or bathing	1.38 (1.34,1.42)	**< 0.001**	1.35 (1.29,1.42)	**< 0.001**
Doing errands alone	1.43 (1.40,1.47)	**< 0.001**	1.38 (1.33,1.44)	**< 0.001**

Note: OR = unadjusted odds ratio; aOR = adjusted odds ratio; CI = confidence interval; p-values < 0.05 denote statistical significance.

### 3.6 Temporal changes in the association between depressive symptoms and overall disability

**Fig. 2:**
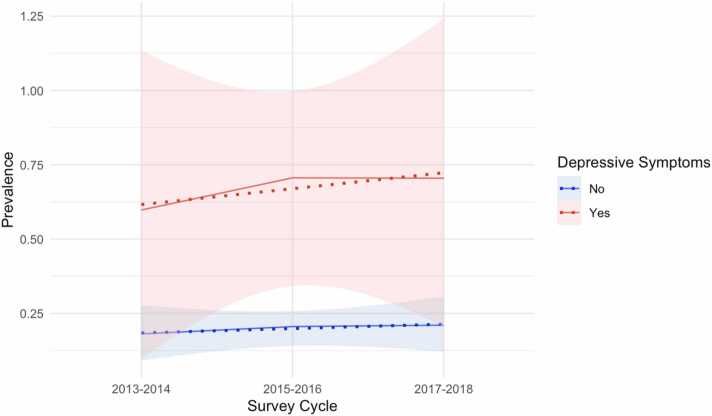
Mean trajectory of limitations in overall disability prevalence by depressive symptoms.

Across 2013 to 2018, individuals with depressive symptoms consistently showed a higher prevalence of disability compared to those without depressive symptoms (Fig. 2). In participants with depressive symptoms, the prevalence of disability showed a gradual increase over the survey cycles. In contrast, participants without depressive symptoms demonstrated a relatively stable prevalence of disability across the same time period.

The confidence intervals (shaded areas) around the prevalence estimates highlight greater uncertainty in estimates for those with depressive symptoms, while the trend for individuals without depressive symptoms appear more stable. This difference may reflect the variability in sample size or other unmeasured factors influencing disability outcomes in those with depressive symptoms. These findings underscore the strong association between depressive symptoms and disability and suggest that this association has persisted and even slightly intensified over time.

## 4 Discussion

The results of our study demonstrated a clear link between depressive symptoms and overall disability. Individuals experiencing depressive symptoms were significantly more likely to report various disability-related limitations compared to those without depressive symptoms. Specifically, these symptoms correlated with heightened odds of difficulties across multiple domains, including hearing, seeing, concentrating, walking, dressing, and completing errands independently. The findings further revealed that both cognitive-affective and somatic symptoms contributed to overall disability and specific difficulties. Each incremental increase in cognitive-affective and somatic symptom scores was linked to a greater likelihood of encountering functional impairments. Over the surveyed time period, the prevalence of disability among individuals with depressive symptoms had increased, while those without depressive symptoms experienced relatively stable disability levels.

The findings of our study align with existing literature demonstrating an association between depression and disability [13], [14], [15], [16]. Previous research, including a meta-analysis of 41 studies from 22 countries, has established a broad connection between depressive symptoms and disability across diverse populations [16]. While this meta-analysis highlighted a general link, our study contributes to this field by utilizing the NHANES dataset to investigate the association at a national level in the US, with a specific focus on distinct disability domains. Our study differs from previous research by examining six specific disability domains, allowing for a more precise assessment of how depressive symptoms relate to various aspects of daily functioning. This approach reveals differences in the strength of association between depression and specific functional limitations, providing a more detailed understanding of the impact of depression on disability. Additionally, our study incorporated a temporal analysis, indicating that the relationship between depressive symptoms and disability may intensify over time. By employing standardized assessments, including the PHQ-9 and a detailed disability assessment, our study offers a more consistent evaluation of depressive symptoms and disability compared to studies that use varied assessment tools. These methodological strengths allow our study to not only support but also refine previous findings, contributing a targeted and longitudinal perspective on the relationship between depressive symptoms and specific disability domains within the United States population.

Depressive symptoms, along with both cognitive-affective and somatic scores, were most strongly associated with difficulties in concentrating. Previous studies have extensively indicated the association between depression and concentration problems and cognitive impairment [17], [18], [19]. These concentration difficulties may stem from disruptions in attention, memory, and executive function, which are often impaired in depressive states [20]. Neurobiologically, this may be linked to altered activity in the prefrontal cortex and hippocampus, regions involved in cognitive processing [21]. Additionally, heightened stress and fatigue, common in depression, may further exacerbate cognitive impairments, making concentration one of the most affected domains [22]. The strong association of cognitive-affective and somatic scores with concentration difficulties reflects how both emotional and physical symptoms of depression disrupt cognitive function. Cognitive-affective symptoms, such as persistent negative thoughts, rumination, and emotional dysregulation, interfere with the brain’s ability to focus and process information [23]. Somatic symptoms, such as fatigue and sleep disturbances, further impair cognitive resources, leading to greater difficulties in concentration [24]. Together, these overlapping effects of emotional and physical symptoms contribute to the pronounced impact on cognitive function, particularly in tasks requiring sustained attention and mental effort.

Hearing was the least affected domain in its association with depression, cognitive-affective, and somatic scores, likely because auditory function is primarily a sensory process that remains relatively intact even in the presence of mental health disorders [25]. Unlike concentration, which directly involves cognitive and emotional processing, hearing is more dependent on the peripheral and central auditory systems, which are less influenced by the neurobiological changes seen in depression [26]. Moreover, while depression can affect sensory perception to some extent, hearing loss is generally associated with aging, noise exposure, or medical conditions rather than mood or cognitive symptoms [27], [28]. The mechanisms of depression, such as dysregulation of neurotransmitters and structural brain changes, tend to impact regions of the brain associated with cognition, emotion, and motor control, rather than the auditory pathways [29]. Thus, depressive symptoms may influence one’s perception or interpretation of sensory input (such as increased sensitivity to noise), but it is less likely to directly impair hearing ability itself, explaining the weaker association in this domain.

Our findings on the temporal association between depressive symptoms and disability revealed a consistently higher prevalence of disability among individuals with depressive symptoms compared to those without. Over the survey cycles, participants with depressive symptoms showed a gradual increase in disability prevalence, while those without depressive symptoms demonstrated a more stable trend. This suggests that the relationship between depression and disability may have intensified over time. The greater variability in prevalence estimates for those with depressive symptoms may indicate unmeasured factors such as comorbidities or socioeconomic influences affecting disability outcomes. These results underscore the persistent and potentially worsening impact of depressive symptoms on functional impairments, highlighting the critical need for addressing mental health to mitigate disability risk.

Our study has both strengths and limitations. Among its strengths, the use of the NHANES dataset provides a representative and comprehensive sample of the U.S. population. Additionally, the diverse data collection methods, including interviews and surveys conducted by trained professionals, contribute to the robustness of the findings. However, there are several limitations. First, the study’s cross-sectional design limits our ability to infer causality or observe changes in the association between depressive symptoms and disability over time. Furthermore, the NHANES dataset relies on self-reported data, which may introduce bias, such as social desirability or recall bias. An additional limitation is that we cannot determine whether disabilities, such as sensory impairments (hearing or vision loss) or cognitive decline, preceded depressive symptoms or were consequences of them. In some cases, depression could be an early manifestation of an underlying impairment. Despite these limitations, our study provides valuable insights into the complex relationship between depressive symptoms and disability.

This study contributes to the understanding of the relationship between depressive symptoms and disability, demonstrating that depressive symptoms are strongly associated with both overall and domain-specific disabilities. The findings highlight the need for extensive approaches to managing depression that address both mental health and functional impairment, and suggest that the impact of depressive symptoms on disability may increase over time. Future research should explore causal relationships and develop targeted interventions to alleviate the impact of depressive symptoms on disability.

## Funding

This research did not receive any specific grant from funding agencies in the public, commercial, or not-for-profit sectors.

## Declaration of Competing Interest

The authors declare the following financial interests/personal relationships which may be considered as potential competing interests: Venkat Bhat reports financial support was provided by Academic Scholar Award from the University of Toronto Department of Psychiatry. Venkat Bhat reports financial support was provided by Research support from the Canadian Institutes of Health Research, Brain & Behavior Foundation, Ontario Ministry of Health Innovation Funds, Royal College of Physicians and Surgeons of Canada. Venkat Bhat reports was provided by Department of National Defense (Government of Canada), New Frontiers in Research Fund, Associated Medical Services Inc Healthcare, American Foundation for Suicide Prevention, Roche Canada, Novartis, and Eisai. VB is supported by an Academic Scholar Award from the University of Toronto Department of Psychiatry and has received research support from the Canadian Institutes of Health Research, Brain & Behavior Foundation, Ontario Ministry of Health Innovation Funds, Royal College of Physicians and Surgeons of Canada, Department of National Defense (Government of Canada), New Frontiers in Research Fund, Associated Medical Services Inc Healthcare, American Foundation for Suicide Prevention, Roche Canada, Novartis, and Eisai. If there are other authors, they declare that they have no known competing financial interests or personal relationships that could have appeared to influence the work reported in this paper.
