# Supplementary material for: The association between depressive symptoms and limitations in disability domains among US adults
Source: J Mood Anxiety Disord. 2024 Dec 30;9:100103. doi: 10.1016/j.xjmad.2024.100103 (PMC12244210; doi:10.1016/j.xjmad.2024.100103)
Supplement: Supplementary file 1 — Supplementary material [file mmc1.docx]

**Supplementary materials**

**Table S1.** Main effects models for the associations of total PHQ-8 scores with each domain of disability.

| **PHQ-8 Scores** | | | | |
| --- | --- | --- | --- | --- |
|  | OR  (95% CI) | *p*-value | aOR  (95% CI) | *p*-value |
| Hearing | 1.09  (1.07,1.11) | **<0.001** | 1.09  (1.07,1.12) | **<0.001** |
| Seeing | 1.14  (1.13,1.16) | **<0.001** | 1.11  (1.09,1.14) | **<0.001** |
| Concentrating | 1.28  (1.26,1.30) | **<0.001** | 1.26  (1.23,1.29) | **<0.001** |
| Walking | 1.19  (1.17,1.20) | **<0.001** | 1.17  (1.15,1.19) | **<0.001** |
| Dressing or bathing | 1.20  (1.18,1.23) | **<0.001** | 1.19  (1.16,1.22) | **<0.001** |
| Doing errands alone | 1.24  (1.22,1.27) | **<0.001** | 1.22  (1.19,1.25) | **<0.001** |

Note: OR = unadjusted odds ratio; aOR = adjusted odds ratio; CI = confidence interval; *p*-values < 0.05 denote statistical significance.
